# Tandem Aldol-Michael reactions in aqueous diethylamine medium: a greener and efficient approach to dimedone-barbituric acid derivatives

**DOI:** 10.1186/1752-153X-8-9

**Published:** 2014-02-01

**Authors:** Assem Barakat, Abdullah Mohammed Al-Majid, Abdulaziz Moshabab Al-Ghamdi, Yahia Nasser Mabkhot, Mohammed Rafiq Hussain Siddiqui, Hazem A Ghabbour, Hoong-Kun Fun

**Affiliations:** 1 Department of Chemistry, College of Science, King Saud University, P.O. Box 2455, Riyadh 11451, Saudi Arabia; 2 Department of Chemistry, Faculty of Science, Alexandria University, P.O. Box 426, Ibrahimia, Alexandria 21321, Egypt; 3 Department of Pharmaceutical Chemistry, College of Pharmacy, King Saud University, P.O. Box 2457, Riyadh 11451, Saudi Arabia

**Keywords:** Tandem Aldol-Michael reactions, MCRs, Barbituric acid, Aqueous media, Green chemistry, Dimedone, Zwitterions

## Abstract

**Background:**

Green chemistry is a rapidly developing new field that provides us with a proactive avenue for the sustainable development of future science and technologies. Green chemistry uses highly efficient and environmentally benign synthetic protocols to deliver lifesaving medicines, accelerating lead optimization processes in drug discovery, with reduced unnecessary environmental impact. From this view point, it is desirable to use water instead of organic solvents as a reaction medium, since water is safe, abundant and an environmentally benign solvent.

**Results:**

A convenient one-pot method for the efficient synthesis of the novel Zwitterion derivatives **4a-p***via* a three-component condensation reaction of barbituric acid derivatives **1a,b**, dimedone **2**, and various aldehydes **3** in the presence of aqueous diethylamine media is described. This new approach is environmentally benign, with clean synthetic procedure, short reaction times and easy work-up procedure which proceeded smoothly to provide excellent yield (88-98%). The synthesized products were characterized by elemental analysis, IR, MS, NMR and CHN analysis. The structure of **4a** was further confirmed by single crystal X-ray diffraction. The compound crystallizes in the orthorhombic space group *Pbca* with *α* = 14.6669 (5) Å, *b* = 18.3084 (6) Å, *c* = 19.0294 (6) Å, α = 90°, *β* = 90°, = 90°, *V* = 5109.9 (3) Å^3^, and *Z* = 8. The molecules are packed in crystal structure by weak intermolecular C–H⋅ ⋅ ⋅O hydrogen bonding interactions.

**Conclusions:**

An environmentally benign Aldol-Michael protocol for the synthesis of dimedone-barbituric derivatives using aqueous diethylamine medium is achieved.

## Background

Recently, the development of environmentally benign and clean synthetic procedures has become the goal of organic synthesis. Water plays an essential role in life processes and also as a medium for organic reactions [1,2]. The use of water as a reaction medium exhibits remarkable benefit because of its high polarity and therefore immiscibility with most organic compounds. Reactions in aqueous media are environmentally safe and have less carcinogenic effects with a simple work up procedure which are especially important in industry. Thus, there is a need for developing multicomponent reactions (MCR’s) in water, without the use of any harmful organic solvents.

**Figure 1:**
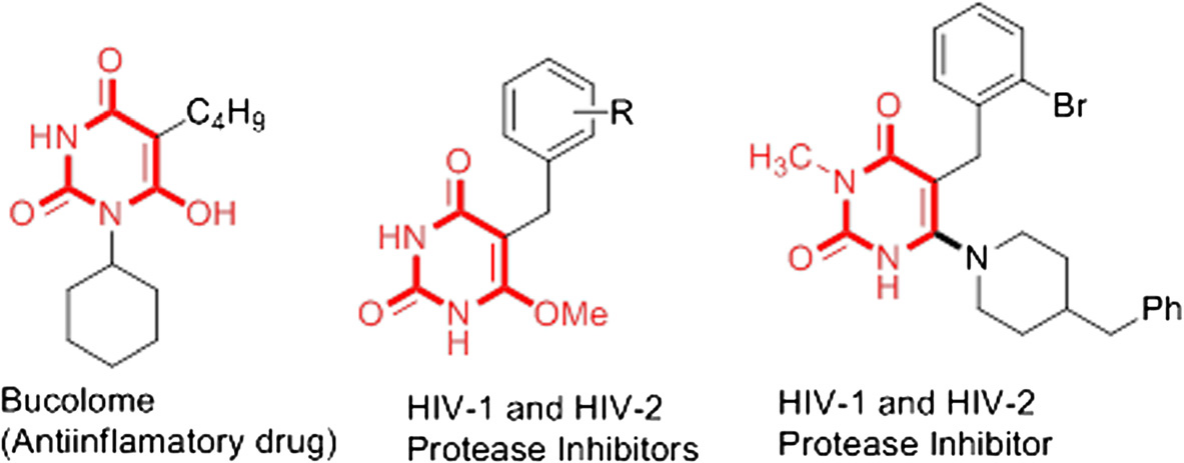
Bioactive compounds containing the barbituric acid framework.

On the other hand, due to the diverse biological properties of barbituric acid derivatives (**1**), there is a widespread interest in their synthesis [3-7]. Compounds alkylated in the fifth position have demonstrated anticancer, HIV-1 and HIV-2 protease inhibitors [8], sedative-hypnotic [9,10] and anticonvulsant [11] properties. Many of their representatives have clinical use as anti-inflammatory [12] and hypnotic drugs, such as veronal, phenobarbital, seconal, bucolone and sodium pentothal (Figure 1) [13-15]. A number of compounds having these systems have been synthesized with diverse pharmacological activities [16,17].

Dimedone (5,5-dimethylcyclohexane-1,3-dione) **2** belongs to the cyclic 1,3-diketones – a very important class of organic compounds. A wide range of practical applications of dimedone include their uses as versatile precursors for synthesis of numerous hetero and spirocyclic compounds [18], xanthene derivatives with their industrial [19] and synthetic [20] applications, and also as reagent for various analytical determinations [21].

As a part of our work on one-pot multicomponent reactions (MCRs) for the synthesis of various heterocyclic compounds, we report here a highly efficient procedure for the preparation of dimedone-barbituric derivatives based on tandem Aldol-Michael reactions using aqueous diethylamine medium.

## Results and discussion

**Scheme 1:**
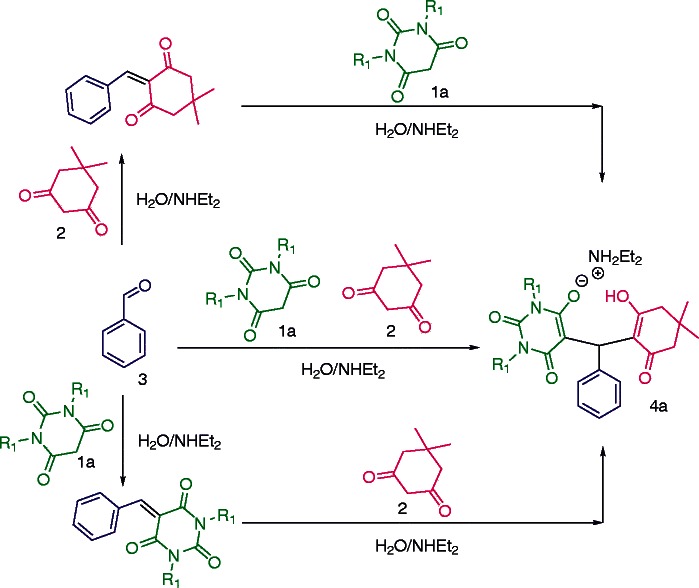
Synthesis of 4a.

In a typical experimental procedure, a mixture of barbituric acid **1a,b**, dimedone **2** and aromatic aldehyde **3** in water was stirred in the presence of a stoichiometric amount of diethylamine (1.0 equiv.) to afford the ‘Zwitterion adduct salts’ of dimedone-barbituric acid derivative **4a** in high yields (Scheme 1).

**Figure 2:**
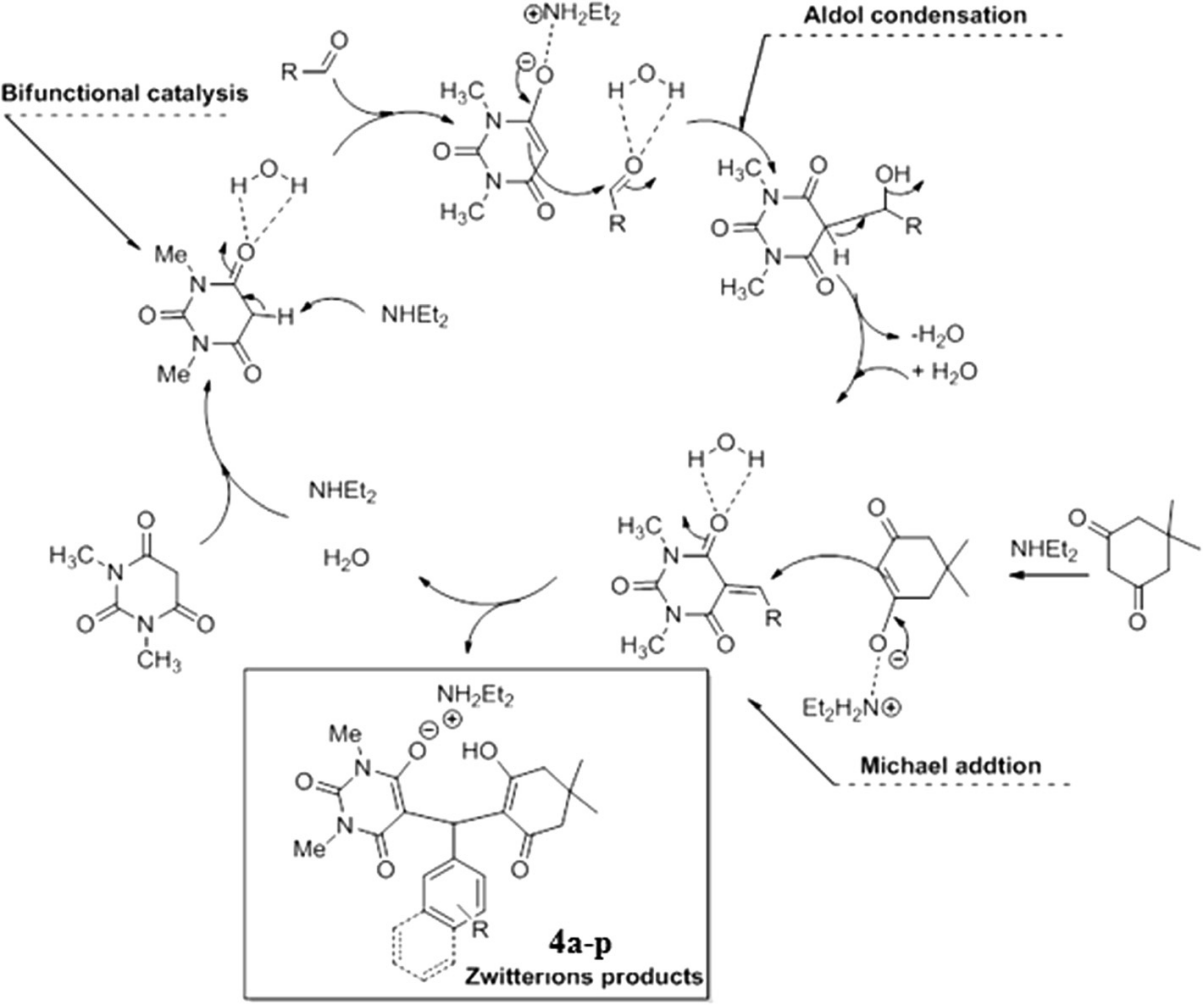
A possible mechanistic pathway.

A possible mechanism for the tandem Aldol- Michael reaction is shown in Figure 2. In the first step of the reaction, olefin is produced by a Aldol condensation between aryl aldehyde **3** and **1a,b** promoted by DEA. Dimedone in the presence of DEA is then converted to its corresponding diethylammonium dimedonate that easily reacts with olefin to give product **4a-p**[22-31].

In the absence of DEA, the reaction does not proceed efficiently and only a poor yield of products was obtained after 10 h. The structures of products were confirmed by physical and spectroscopic (IR, MS, NMR) data, and by elemental analysis. The workup procedure is very simple and the products do not require further purification.

**Figure 3:**
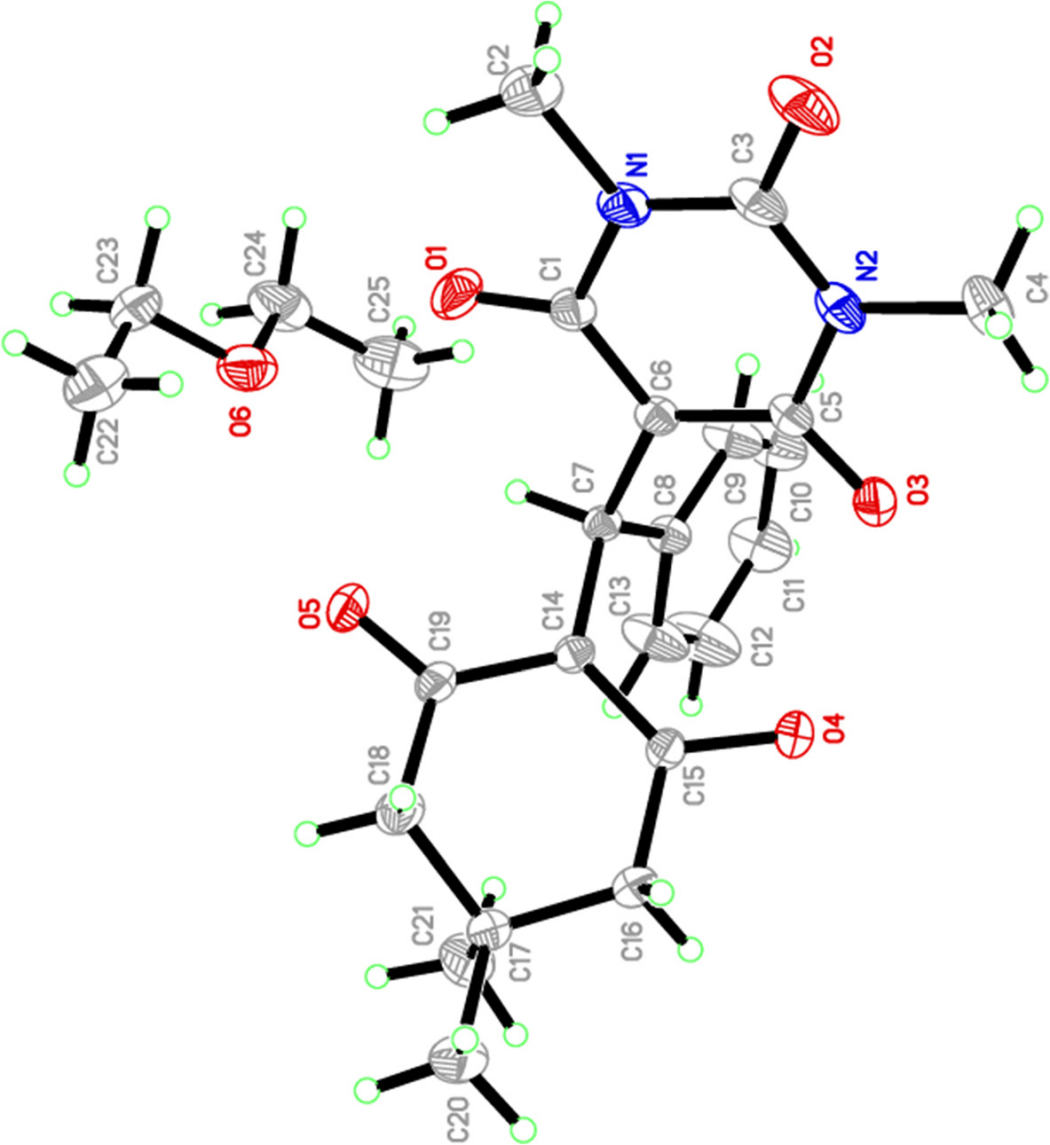
ORTEP representation of the structure of 4a.

**Figure 4:**
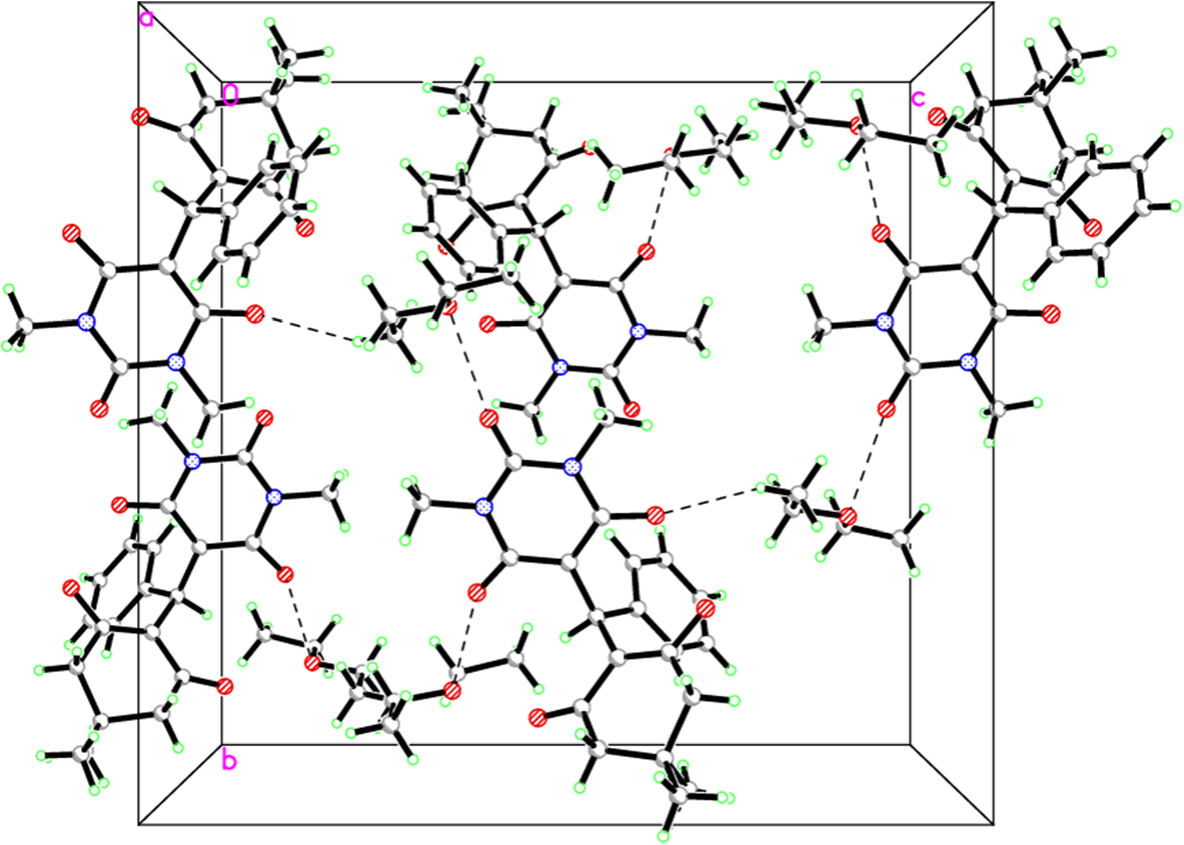
**Crystal packing showing intermolecular C–H⋅O hydrogen bonds as dashed lines. 4a**.

The X-ray zwitterion structure of **4a** (Figure 3) was obtained using X-ray structure determination from a single crystal grown from CHCl_3_/Et_2_O as solvents. The structure shows interesting characteristics (Table 1). We were unable to determine the location of the C6 and C14 hydrogens by ^1^HNMR analysis. This is because the hydrogen from C6 dimedone, rather than hydrogen from C14 of the barbituric acid moiety, is removed by the basicity of diethylamine. This was confirmed by the X-ray structure because one hydrogen is on the diethylamine and the other is involved in hydrogen bonding interactions between both barbituric acid and dimedone moiety. The hydrogen-bonding interactions are listed in Table 2. Figure 4 depicts the packing of the molecules in the crystal structure. The crystal structure is stabilized by C–H⋅ ⋅ ⋅O hydrogen bonds into a three-dimensional framework structure. It is noteworthy to mention that ^1^HNMR have also shown a singlet signal at *δ* 15.28 ppm which can be assigned to the OH group which makes a hydrogen bond.

**Table 1:** Crystallographic data and refinement information of 4a

**Empirical formula**	**C**_**25**_**H**_**32**_**N**_**2**_**O**_**6**_
Formula weight	456.53
Temperature (K)	293
Crystal system	Orthorhombic
Space group	*Pbca*
Cu Kα radiation, λ	1.54178 Å
*a* =	14.6669 (5) Å
*b* =	18.3084 (6) Å
*c* =	19.0294 (6) Å
α =	90º
β =	90º
γ =	90º
*V* =	5109.9 (3) Å^3^
*Z* =	8
Theta range for data collection	3.0–69.2°
μ =	0.70 mm^−1^
Density clac. (g/cm^3^)	1.187
Crystal shape and colour	Plate, colourless
Crystal size	0.89 × 0.78 × 0.22 mm
h/k/l	−17,17/-22,22/-22,23
Measured reflections	32924
Independent reflections	4796 (*R*_int_ = 0.088)
Reflections with *I* > 2σ(*I*)	3997
Goodness-of-fit on *F*^*2*^	1.04
*R*[*F*^2^ > 2σ(*F*^2^)] =	0.067
*wR*(*F*^2^) =	0.195
Δρ_max_ =	0.47 e Å^−3^
Δρ_min_ =	−0.40 e Å^−3^

**Table 2:** Hydrogen-bond geometry (Å, °)

***D*—H · · ·** ***A***	***D*—H**	**H · · ·** ***A***	***D*** **· · ·** ***A***	***D*—H · · ·** ***A***
C2—H2B · · · O1	0.9600	2.2600	2.655(3)	104.00
C4—H4B · · · O3	0.9600	2.2300	2.682(3)	108.00
C7—H7A · · · O1	0.9800	2.3700	2.894(2)	113.00
C7—H7A · · · O5	0.9800	2.2800	2.821(2)	114.00
C22—H22A · · · O3^i^	0.9600	2.5400	3.376(3)	146.00

Symmetry code: (i) *x*, −*y* + 3/2, *z* + 1/2.

**Scheme 2:**
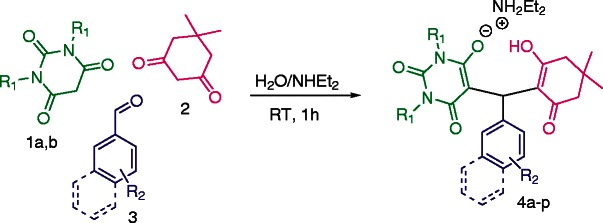
Synthesis of 4a-p.

With the optimal reaction conditions established, the generality of the Aldol-Michael reactions was next investigated by using a series of aryl aldehyde **3** (Table 3). Various aldehydes derivatives with either electron-withdrawing or electron-donating groups at the *para*-, *meta*-, or even sterically hindered *ortho*-position on the aromatic ring were tolerated and gave the corresponding condensed products **4a-p** in excellent chemical yield up to 98% (Scheme 2).

**Table 3:** **Tandem Aldol-Michael reactions of barbituric acid 1a,b and dimedone 2 with aldehydes 3 in aqueous diethylamine medium**^***a***^

**#**	**3**	**R**_**1**_	**R**_**2**_	**yield (%)**^***b***^
1	**4a**	CH_3_	Ph	98
2	**4b**	CH_3_	*p*-CH_3_Ph	97
3	**4c**	CH_3_	*p*-ClPh	97
4	**4d**	CH_3_	*p*-BrPh	95
5	**4e**	CH_3_	*m*-BrPh	93
6	**4f**	CH_3_	*p*-CH_3_OPh	92
7	**4 g**	CH_3_	*o*-NO_2_Ph	93
8	**4 h**	CH_3_	2,4-Cl_2_Ph	90
9	**4i**	CH_3_	2,6-Cl_2_Ph	89
10	**4j**	CH_3_	2-Naphthaldehyde	94
11	**4 k**	CH_3_	*p*-HO-Ph	91
12	**4 l**	H	Ph	93
13	**4 m**	H	*p*-CH_3_Ph	91
14	**4n**	H	*p*-ClPh	90
15	**4o**	H	*p*-BrPh	89
16	**4p**	H	2-Naphthaldehyde	90

^a^All reactions were carried out with barbituric acid derivatives **1a,b** (1.5 mmol), dimedone **2** (1.5 mmol) aldehydes **3** (1.5 mmol) and diethylamine (1.5 mmol) in water (1.5 mL) for the specified time. ^b^Yield of isolated product **4a-p**.

## Conclusions

In summary, a mild, efficient, and expeditious method has been developed for the synthesis of zwitterion-condensed products **4a-p***via* a three component; one-pot cyclocondensation reaction of aromatic aldehyde, barbituric acid, and dimedone using aqueous diethylamine medium. The main advantage of the present methodology is a simple work-up procedure with milder reaction conditions. This method provides excellent yields of the products with high selectivity. Further studies on expanding the application of this method and the biological evaluation of these dimedone-barbituric derivatives are in progress.

### Experimental section

#### General

All chemicals were purchased from Aldrich, Sigma-Aldrich, Fluka etc., and were used without further purification, unless otherwise stated. All melting points were measured on a Gallenkamp melting point apparatus in open glass capillaries and are uncorrected. IR Spectra were measured as KBr pellets on a Nicolet 6700 FT-IR spectrophotometer. The NMR spectra were recorded on a Jeol-400 NMR spectrometer. ^1^H NMR (400 MHz), and ^13^C NMR (100 MHz) were run in either deuterated dimethylsulphoxide (DMSO-*d*_6_) or deuterated chloroform (CDCl_3_). Chemical shifts (*δ*) are referred in terms of *ppm* and *J* -coupling constants are given in *Hz*. Mass spectra were recorded on a Jeol of JMS-600H. Elemental analysis was carried out on an Elmer 2400 Elemental Analyzer; CHN mode.

#### General procedure for aldol condensation Michael addition for the synthesis of 4a-p (GP1)

A mixture of aldehyde **3** (1.5 mmol), dimedone **2** (1.5 mmol), barbituric acid derivatives **1a,b** (1.5 mmol) and Et_2_NH (1.5 mmol, 155 μL) in 1.5 mL of degassed H_2_O was stirred at room temperature for 1–2 hours until TLC showed complete disappearance of the reactants. The product precipitated and the mixture was filtered and washed with ether (3 × 20 mL). The solid was recrystallized from a mixture of CH_2_Cl_2_/Et_2_O to afford pure product **4a-p**.

#### 5-((2-Hydroxy-4,4-dimethyl-6-oxocyclohex-1-en-1-yl)(phenyl)methyl)-1,3-dimethyl-2,6-dioxo-1,2,3,6-tetrahydropyrimidin-4-olate (4a)

**4a** was prepared from 1,3-dimethylbarbituric acid **1a**, dimedone **2** and benzaldehyde according to the general procedure (**GP1**) yielding colorless crystalline material (671 mg, 1.47 mmol, 98%). m.p: 159°C; IR (KBr, *cm*^*–1*^): 3150, 2959, 1667, 1617, 1585, 1422, 1256, 1227; ^1^H NMR (400 MHz, CDCl_3_): *δ* 15.28 (s, 1H, OH), 7.17-7.04 (m, 5H, Ph), 5.85 (s, 1H, benzyl-H), 3.29 (s, 12H, 4CH_3_), 2.96 (q, 4H, *J* = 7.3 Hz, C*H*_2_CH_3_), 2.42 (d, 2H, *J* = 5.1 Hz, CH_2_), 2.29 (m, 2H, CH_2_), 1.24 (t, 6H, *J* = 7.3 Hz, CH_2_C*H*_3_), 1.14 (s, 3H, CH_3_), 1.05 (s, 3H, CH_3_); ^13^C NMR (100 MHz, CDCl_3_): *δ* = 192.5, 180.8, 152.5, 142.5, 128.0, 126.7, 125.1, 116.3, 90.9, 51.4, 45.9, 42.2, 33.0, 31.5, 29.6, 28.4, 27.6, 11.4; LC/MS (ESI): 457 [M]^+^; Anal. for C_25_H_35_N_3_O_5_; calcd: C, 65.62; H, 7.71; N, 9.18; Found: C, 65.61; H, 7.73; N, 9.20.

The structure of **4a** was confirmed by X-ray crystal structure analysis. CCDC- 933624 contains the supplementary crystallographic data for this compound. This data can be obtained free of charge from the Cambridge Crystallographic Data Centre *via*http://www.ccdc.cam.ac.uk/data_request/cif. A colorless crystal suitable for X-ray analysis was obtained from recrystallization of the compound from CHCl_3_/Et_2_O at room temperature after 2 days.

#### 5-((2-Hydroxy-4,4-dimethyl-6-oxocyclohex-1-en-1-yl)(p-tolyl)methyl)-1,3-dimethyl-2,6-dioxo-1,2,3,6-tetrahydropyrimidin-4-olate (4b)

**4b** was prepared from 1,3-dimethylbarbituric acid **1a**, dimedone **2** and p-tolualdehyde according to the general procedure (**GP1**) yielding an oily material (685 mg, 1.45 mmol, 97%). IR (KBr, *cm*^*–1*^): 3150, 2954, 2867, 1675, 1580, 1508, 1447, 1380, 1256, 1145; ^1^H NMR (400 MHz, CDCl_3_): *δ* 15.25 (s, 1H, OH), 7.00-6.93 (m, 4H, Ph), 5.84 (s, 1H, benzyl-H), 3.28 (s, 12H, 4CH_3_), 2.90 (q, 4H, *J* = 7.3 Hz, C*H*_2_CH_3_), 2.30 (d, 4H, *J* = 5.1 Hz, CH_2_), 2.22 (s, 3H, CH_3_), 1.20 (t, 6H, *J* = 7.3 Hz, CH_2_C*H*_3_), 1.16 (s, 3H, CH_3_), 1.04 (s, 3H, CH_3_); ^13^C NMR (100 MHz, CDCl_3_): *δ* = 196.5, 180.1, 152.8, 140.5, 134.2, 129.8, 128.7, 126.8, 126.7, 115.6, 91.0, 51.4, 45.9, 42.5, 32.6, 31.5, 29.6, 28.4, 27.6, 20.9, 11.9; LC/MS (ESI): 471 [M]^+^; Anal. for C_26_H_37_N_3_O_5_; calcd: C, 66.22; H, 7.91; N, 8.91; Found: C, 66.24; H, 7.92; N, 8.87.

#### 5-((4-Chlorophenyl)(2-hydroxy-4,4-dimethyl-6-oxocyclohex-1-en-1-yl)methyl)-1,3-dimethyl-2,6-dioxo-1,2,3,6-tetrahydropyrimidin-4-olate (4c)

**4c** was prepared from 1,3-dimethylbarbituric acid **1a**, dimedone **2** and *p*-chlorobenzaldehyde **3** according to the general procedure (**GP1**) yielding an oily material (715 mg, 1.45 mmol, 97%). IR (KBr, *cm*^*–1*^): 3151, 2955, 2868, 2497, 1675, 1580, 1481, 1444, 1379, 1258, 1206; ^1^H NMR (400 MHz, CDCl_3_): *δ* 15.02 (s, 1H, OH), 7.12-6.95(m, 4H, Ph), 5.87 (s, 1H, benzyl-H), 3.30 (s, 12H, 4CH_3_), 2.90 (q, 4H, *J* = 7.3 Hz, C*H*_2_CH_3_), 2.38 (s, 4H, CH_2_), 1.20 (t, 6H, *J* = 7.3 Hz, CH_2_C*H*_3_), 1.16 (s, 3H, CH_3_), 1.04(s, 3H, CH_3_); ^13^C NMR (100 MHz, CDCl_3_): *δ* = 198.1, 181.0, 152.5, 141.5, 130.6, 128.3, 128.2, 128.0, 127.9, 115.2, 90.7, 65.9, 49.8, 42.3, 32.4, 31.5, 31.2, 29.6, 28.4, 27.6, 15.3, 11.4; LC/MS (ESI): 492 [M]^+^; Anal. for C_25_H_34Cl_N_3_O_5_; calcd: C, 61.03; H, 6.97; Cl, 7.21; N, 8.54; Found: C, 61.06; H, 7.00; Cl, 7.18; N, 8.57.

#### 5-((4-Bromophenyl)(2-hydroxy-4,4-dimethyl-6-oxocyclohex-1-en-1-yl)methyl)-1,3-dimethyl-2,6-dioxo-1,2,3,6-tetrahydropyrimidin-4-olate (4d)

**4d** was prepared from 1,3-dimethylbarbituric acid **1a**, dimedone **2** and *p*-bromobenzaldehyde **3** according to the general procedure (**GP1**) yielding an oily material (761 mg, 1.42 mmol, 95%). IR (KBr, *cm*^*–1*^): 3155, 2955, 2867, 2500, 1674, 1579, 1430, 1376, 1204;^1^H NMR (400 MHz, CDCl_3_): *δ* 15.20 (s, 1H, OH), 7.34 (d, 2H, *J* = 8.0 Hz, Ph), 6.98 (d, 2H, *J* = 8.0 Hz, Ph), 5.79 (s, 1H, benzyl-H), 3.27 (s, 12H, 4CH_3_), 2.99 (q, 4H, *J* = 7.3 Hz, C*H*_2_CH_3_), 2.40 (d, 2H, *J* = 5.1 Hz, CH_2_), 2.28 (m, 2H, CH_2_), 1.29 (t, 6H, *J* = 7.3 Hz, CH_2_C*H*_3_), 1.18 (s, 3H, CH_3_), 1.04 (s, 3H, CH_3_); ^13^C NMR (100 MHz, CDCl_3_): *δ* = 199.1, 191.2, 164.8, 152.4, 142.8, 132.5, 131.0, 129.9, 128.7, 128.6, 118.9, 115.9, 90.6, 51.2, 45.8, 42.3, 32.7, 31.5, 29.5, 28.5, 28.3, 27.6, 11.4; LC/MS (ESI): 536 [M]^+^; Anal. for C_25_H_34_BrN_3_O_5_; calcd: C, 55.97; H, 6.39; Br, 14.89; N, 7.83; Found: C, 56.00; H, 6.40; Br, 14.86; N, 7.82.

#### 5-((3-Bromophenyl)(2-hydroxy-4,4-dimethyl-6-oxocyclohex-1-en-1-yl)methyl)-1,3-dimethyl-2,6-dioxo-1,2,3,6-tetrahydropyrimidin-4-olate (4e)

**4e** was prepared from 1,3-dimethylbarbituric acid **1a**, dimedone **2** and *m*-bromobenzaldehyde **3** according to the general procedure (**GP1**) yielding an oily material (745 mg, 1.39 mmol, 93%). IR (KBr, *cm*^*–1*^): 3050, 2955, 2868, 2500, 1675, 1581, 1444, 1378, 1255, 1205; ^1^H NMR (400 MHz, CDCl_3_): *δ* 15.63 (s, 1H, OH), 7.22 (d, 1H, *J* = 7.3 Hz, Ph), 7.19 (s, 1H, Ph), 7.07 (d, 1H, *J* = 7.3 Hz, Ph), 7.05 (d, 1H, *J* = 7.3 Hz, Ph), 5.84 (s, 1H, benzyl-H), 3.34 (s, 6H, 2CH_3_), 3.32 (s, 6H, 2CH_3_), 2.98 (q, 4H, *J* = 7.3Hz, CH_2_CH_3_), 2.31 (d, 4H, *J* = 5.1 Hz, CH_2_), 1.24 (t, 6H, *J* = 7.3 Hz, CH_2_C*H*_3_), 1.12 (s, 3H, CH_3_), 1.03 (s, 3H, CH_3_); ^13^C NMR (100 MHz, CDCl_3_): *δ* = 190.8, 186.4, 165.2, 164.4, 151.7, 144.7, 129.7,129.6, 128.7, 125.3, 91.5, 42.1, 34.4, 28.9, 28.7, 11.5; LC/MS (ESI): 536 [M]^+^; Anal. for C_25_H_34_BrN_3_O_5_; calcd: C, 55.97; H, 6.39; Br, 14.89; N, 7.83; Found: C, 56.01; H, 6.41; Br, 14.86; N, 7.84.

#### 5-((2-Hydroxy-4,4-dimethyl-6-oxocyclohex-1-en-1-yl)(4-methoxyphenyl)methyl)-1,3-dimethyl-2,6-dioxo-1,2,3,6-tetrahydropyrimidin-4-olate (4f)

**4f** was prepared from 1,3-dimethylbarbituric acid **1a**, dimedone **2** and anisaldehyde **3** according to the general procedure (**GP1**) yielding an oily material (672 mg, 1.38 mmol, 92%). IR (KBr, *cm*^*–1*^): 3047, 2953, 2866, 2499, 1679, 1577, 1510, 1427, 1373, 1255, 1214;^1^H NMR (400 MHz, CDCl_3_): *δ* 15.26 (s, 1H, OH), 6.98 (d, 2H, *J* = 8.0 Hz, Ph), 6.72 (d, 2H, *J* = 8.0 Hz, Ph), 5.69 (s, 1H, benzyl-H), 3.71 (s, 3H, CH_3_), 3.29 (s, 12H, 4CH_3_), 2.87 (q, 4H, *J* = 7.3 Hz, C*H*_2_CH_3_), 2.31 (d, 4H, *J* = 5.1 Hz, CH_2_), 1.19 (t, 6H, *J* = 7.3 Hz, CH_2_C*H*_3_), 1.12 (s, 3H, CH_3_), 1.03(s, 3H, CH_3_); ^13^C NMR (100 MHz, CDCl_3_): *δ* = 195.1, 187.2, 157.1, 134.5, 133.9, 127.8, 127.6, 115.6, 113.4, 55.2, 42.6, 31.5, 31.1, 27.9, 12.2; LC/MS (ESI): 487 [M]^+^; Anal. for C_26_H_37_N_3_O_6_; calcd: C, 64.05; H, 7.65; N, 8.62;Found: C, 64.11; H, 7.64; N, 8.59.

#### 5-((2-Hydroxy-4,4-dimethyl-6-oxocyclohex-1-en-1-yl)(4-nitrophenyl)methyl)-1,3-dimethyl-2,6-dioxo-1,2,3,6-tetrahydropyrimidin-4-olate (4 g)

**4b** was prepared from 1,3-dimethylbarbituric acid **1a**, dimedone **2** and *p*-nitrobenzaldehyde **3** according to the general procedure (**GP1**) yielding a beige material (700 mg, 1.39 mmol, 93%). m.p: 148°C; IR (KBr, *cm*^*–1*^): 3050, 2950, 2865, 2500, 1669, 1580, 1510, 1427, 1373, 1255, 1214;^1^H NMR (400 MHz, CDCl_3_): *δ* 15.26 (s, 1H, OH), 6.99 (d, 2H, *J* = 8.0 Hz, Ph), 6.72 (d, 2H, *J* = 8.8 Hz, Ph), 5.69 (s, 1H, benzyl-H), 3.71 (s, 12H, 4CH_3_), 2.85 (q, 4H, *J* = 7.3 Hz, C*H*_2_CH_3_), 2.31 (d,4H, *J* = 14.7 Hz, CH_2_), 1.19 (t, 6H, *J* = 7.3 Hz, CH_2_C*H*_3_), 1.12 (s, 3H, CH_3_), 1.03 (s, 3H, CH_3_); ^13^C NMR (100 MHz, CDCl_3_): *δ* = 161.6, 153.2, 145.5, 141.6, 129.1, 128.2, 127.8, 125.8, 88.5, 49.1, 41.9, 27.5, 11.5; LC/MS (ESI): 502 [M]^+^; Anal. for C_25_H_34_N_4_O_7_; calcd: C, 59.75; H, 6.82; N, 11.15; Found: C, 59.73; H, 6.81; N, 11.17.

#### 5-((2,4-Dichlorophenyl)(2-hydroxy-4,4-dimethyl-6-oxocyclohex-1-en-1-yl)methyl)-1,3-dimethyl-2,6-dioxo-1,2,3,6-tetrahydropyrimidin-4-olate (4 h)

**4 h** was prepared from 1,3-dimethylbarbituric acid **1a**, dimedone **2** and 2,4-dichlorobenzaldehyde **3** according to the general procedure (**GP1**) yielding a beige solid material (710 mg, 1.35 mmol, 90%). m.p: 164°C; IR (KBr, *cm*^*–1*^): 3059, 2995, 2867, 2114, 1741, 1658, 1591, 1463, 1429, 1370, 1341, 1256, 1201^1^H-NMR (400 MHz, CDCl_3_): *δ* 14.80 (s, 1H, OH), 7.29 (d, 1H, *J* = 8.0 Hz, Ph), 7.19 (s, 1H, Ph), 7.12 (d, 2H, *J* = 8.0 Hz, Ph), 5.76 (s, 1H, benzyl-H), 3.28 (s, 12H, 4CH_3_), 3.07 (q, 4H, *J* = 7.3 Hz, C*H*_2_CH_3_), 2.37 (s, 2H, CH_2_), 2.27 (d, 2H, *J* = 5.1 Hz, CH_2_), 1.34 (t, 6H, *J* = 7.3 Hz, CH_2_C*H*_3_), 1.04 (s, 3H, CH_3_), 1.01 (s, 3H, CH_3_); ^13^C NMR (100 MHz, CDCl_3_): *δ* = 199.1, 165.4, 164.4, 152.5, 139.8, 133.6, 131.7, 131.2, 129.3, 126.4, 115.7, 89.8, 51.2, 45.7, 41.9, 32.4, 31.2, 28.3, 28.2, 11.3; LC/MS (ESI): 526 [M]^+^; Anal. for C_25_H_33_Cl_2_N_3_O_5_; calcd: C, 57.04; H, 6.32; Cl, 13.47; N, 7.98; Found: C, 57.09; H, 6.31; Cl, 13.44; N, 8.01.

#### 5-((2,6-Dichlorophenyl)(2-hydroxy-4,4-dimethyl-6-oxocyclohex-1-en-1-yl)methyl)-1,3-dimethyl-2,6-dioxo-1,2,3,6-tetrahydropyrimidin-4-olate (4i)

**4i** was prepared from 1,3-dimethylbarbituric acid **1a**, dimedone **2** and 2,6-dichlorobenzaldehyde **3** according to the general procedure (**GP1**) yielding an oily material (702 mg, 1.33 mmol, 89%). IR (KBr, *cm*^*–1*^): 3048, 2955, 2869, 2728, 2494, 1676, 1575, 1428, 1372, 1238, 1196;^1^H NMR (400 MHz, CDCl_3_): *δ* 14.80 (s, 1H, OH), 7.36 (d, 2H, *J* = 8.0 Hz, Ph), 7.29 (t, 1H, *J* = 8.0 Hz, Ph), 7.12 (d, 2H, *J* = 8.0 Hz, Ph), 5.98 (s, 1H, benzyl-H), 3.26 (s, 12H, 4CH_3_), 2.92(q, 4H, *J* = 7.3 Hz, C*H*_2_CH_3_), 2.37 (s, 2H, CH_2_), 2.27 (d, 2H, *J* = 5.1 Hz, CH_2_), 1.24(t, 6H, *J* = 7.3 Hz, CH_2_C*H*_3_), 1.094(s, 3H, CH_3_), 1.04 (s, 3H, CH_3_); ^13^C NMR (100 MHz, CDCl_3_): *δ* = 192.8, 188.9, 165.3, 164.3, 152.5, 149.7, 137.4, 131.5, 129.8, 126.5, 124.2, 115.5, 114.7, 89.9, 53.5, 41.4, 31.9, 28.7, 28.2, 11.4 ; LC/MS (ESI): 526 [M]^+^; Anal. for C_25_H_33_Cl_2_N_3_O_5_; calcd: C, 57.04; H, 6.32; Cl, 13.47; N, 7.98; Found: C, 57.08; H, 6.30; Cl, 13.45; N, 8.00.

#### 5-((2-Hydroxy-4,4-dimethyl-6-oxocyclohex-1-en-1-yl)(naphthalen-2-yl)methyl)-1,3-dimethyl-2,6-dioxo-1,2,3,6-tetrahydropyrimidin-4-olate (4j)

**4j** was prepared from 1,3-dimethylbarbituric acid **1a**, dimedone **2** and 2-naphthaldehyde **3** according to the general procedure (**GP1**) yielding a white solid material (715 mg, 1.41 mmol, 94%). m.p: 170 °C; IR (KBr, *cm*^*–1*^): 2994, 2948, 2866, 2506, 1742, 1651, 1603, 1570, 1526, 1473, 1431, 1362, 1245;^1^H NMR (400 MHz, CDCl_3_): *δ* 14.26 (s, 1H, OH), 7.46-7.22 (m, 7H, naphthyl), 6.20 (s, 1H, benzyl-H), 3.26 (s, 6H, 2CH_3_), 3.23 (s, 6H, 2CH_3_), 3.14 (q, 4H, *J* = 7.3 Hz, C*H*_2_CH_3_), 2.41 (q, 4H, *J* = 5.1 Hz, CH_2_), 2.23 (s, 2H, CH_2_), 1.37 (t, 6H, *J* = 7.3 Hz, CH_2_C*H*_3_), 1.07 (s, 3H, CH_3_), 1.01 (s, 3H, CH_3_); ^13^C NMR (100 MHz, CDCl_3_): *δ* = 199.0, 180.5, 165.3, 164.3, 152.5, 149.7, 136.8, 131.5, 129.9, 126.5, 124.2, 115.5, 114.7, 89.9, 50.9, 45.5, 41.7, 31.3, 30.7, 28.2, 11.1; LC/MS (ESI): 507 [M]^+^; Anal. for C_29_H_37_N_3_O_5_; calcd: C, 68.62; H, 7.35; N, 8.28; Found: C, 68.65; H, 7.34; N, 8.30.

#### 5-((2-Hydroxy-4,4-dimethyl-6-oxocyclohex-1-en-1-yl)(4-hydroxyphenyl)methyl)-1,3-dimethyl-2,6-dioxo-1,2,3,6-tetrahydropyrimidin-4-olate (4 k)

**4 k** was prepared from 1,3-dimethylbarbituric acid **1a**, dimedone **2** and *p*-hydroxybenzaldehyde **3** according to the general procedure (**GP1**) yielding a white solid material (645 mg, 1.36 mmol, 91%). m.p: 162°C; IR (KBr, *cm*^*–1*^): 23097, 2939, 2884, 2828, 2498, 1747, 1574, 1530, 1506, 1466, 1384, 1241;^1^H NMR (400 MHz, DMSO-*d*_6_): *δ* 14.52 (s, 1H, OH), 8.50 (brs, 1H, OH), 6.76 (d, 2H, *J* = 8.0 Hz, Ph), 6.50 (d, 2H, *J* = 8.0 Hz, Ph), 6.04 (s, 1H, benzyl-H), 3.07 (s, 12H, 2CH_3_), 3.14 (q, 4H, *J* = 7.3 Hz, C*H*_2_CH_3_), 2.92 (q, 4H, *J* = 13.9 Hz, CH_2_), 206 (s, 4H, CH_2_), 1.12 (t, 6H, *J* = 7.3 Hz, CH_2_C*H*_3_), 0.98 (s, 3H, CH_3_); ^13^C NMR (100 MHz, DMSO-*d*_6_): *δ* = 198.0, 188.5, 154.1, 136.6, 128.3, 115.3, 114.3, 90.1, 50.9, 45.5, 42.1, 31.6, 30.7, 29.7, 11.7; LC/MS (ESI): 473 [M]^+^; Anal. for C_25_H_35_N_3_O_6_; calcd: C, 63.41; H, 7.45; N, 8.87; Found: C, 63.40; H, 7.43; N, 8.85.

#### 5-((2-Hydroxy-4,4-dimethyl-6-oxocyclohex-1-en-1-yl)(phenyl)methyl)-2,6-dioxo-1,2,3,6-tetrahydropyrimidin-4-olate (4 l)

**4 m** was prepared from barbituric acid **1b**, dimedone **2** and benzaldehyde **3** according to the general procedure (**GP1**) yielding a white solid material (598 mg, 1.39 mmol, 93%). m.p: 215°C; IR (KBr, *cm*^*–1*^): 3027, 2948, 2867, 2156, 1683, 1593, 1451, 1374, 1291, 1257, 1141^1^H-NMR (400 MHz, CDCl_3_): *δ* 12.26 (s, 1H, OH), 9.31 (brs, 2H, NH), 7.12 (m, 5H, Ph), 5.52 (s, 1H, benzyl-H), 2.99 (q, 4H, *J* = 7.3 Hz, C*H*_2_CH_3_), 2.45 (d, 4H, *J* = 5.1 Hz, CH_2_), 1.24(t, 6H, *J* = 7.3 Hz, CH_2_C*H*_3_), 1.09 (s, 3H, CH_3_), 1.03 (s, 3H, CH_3_); ^13^C NMR (100 MHz, CDCl_3_): *δ* = 198.5, 180.8, 152.5, 142.5, 128.0, 126.7, 125.1, 116.3, 90.9, 51.4, 45.9, 42.2, 33.0, 28.4, 27.6, 11.3; LC/MS (ESI): 429 [M]^+^; Anal. for C_23_H_31_N_3_O_5_; calcd: C, 64.32; H, 7.27; N, 9.78; Found: C, 64.29; H, 7.29; N, 9.80.

#### 5-((2-Hydroxy-4,4-dimethyl-6-oxocyclohex-1-en-1-yl)(p-tolyl)methyl)-2,6-dioxo-1,2,3,6-tetrahydropyrimidin-4-olate (4 m)

**4n** was prepared from barbituric acid **1a**, dimedone **2** and tolualdehyde **3** according to the general procedure (**GP1**) yielding a white solid material (604 mg, 1.36 mmol, 91%). m.p: 213°C; IR (KBr, *cm*^*–1*^): 3150, 2955, 2867, 1690, 1592, 1508, 1375, 1256, 1232, 1167; ^1^H NMR (400 MHz, CDCl_3_): *δ* 13.31 (s, 1H, OH), 8.83 (brs, 2H, NH), 7.27 (d, 2H, *J* = 8.0 Hz, Ph), 7.00 (d, 2H, *J* = 8.0Hz, Ph), 5.88 (s, 1H, benzyl-H), 2.83 (q, 4H, *J* = 7.3 Hz, C*H*_2_CH_3_), 2.31 (d, 4H, *J* = 5.1 Hz, CH_2_), 2.23 (s, 3H, CH_3_), 1.19 (t, 6H, *J* = 7.3 Hz, CH_2_C*H*_3_), 1.04 (s, 3H, CH_3_), 1.02 (s, 3H, CH_3_); ^13^C NMR (100 MHz, CDCl_3_): *δ* = 196.5, 180.1, 152.8, 140.5, 131.4, 130.7, 128.7, 128.6, 118.5, 115.6, 91.0, 50.9, 42.8, 31.6, 31.5, 29.2, 28.3, 27.8, 20.9, 11.3; LC/MS (ESI): 443 [M]^+^; Anal. for C_24_H_33_N_3_O_5_; calcd: C, 64.99; H, 7.50; N, 9.47; Found: C, 64.95; H, 7.49; N, 9.50.

#### 5-((4-Chlorophenyl)(2-hydroxy-4,4-dimethyl-6-oxocyclohex-1-en-1-yl)methyl)-2,6-dioxo-1,2,3,6-tetrahydropyrimidin-4-olate (4n)

**4o** was prepared from barbituric acid **1a**, dimedone **2** and *p*-chlorobenzaldehyde **3** according to the general procedure (**GP1**) yielding an oily product (625 mg, 1.35 mmol, 90%). IR (KBr, *cm*^*–1*^): 3049, 2954, 2865, 2499, 1738, 1699, 1590, 1483, 1375, 1292, 1258, 1225, 1205; ^1^H NMR (400 MHz, CDCl_3_*δ* 13.32 (s, 1H, OH), 8.83 (brs, 2H, NH), 7.27 (d, 2H, *J* = 8.0 Hz, Ph), 7.00 (d, 2H, *J* = 8.0 Hz, Ph), 5.89 (s, 1H, benzyl-H), 2.88 (q, 4H, *J* = 7.3 Hz, C*H*_2_CH_3_), 2.31 (d, 4H, *J* = 5.1 Hz, CH_2_), 1.19 (t, 6H, *J* = 7.3 Hz, CH_2_C*H*_3_), 1.09(s, 3H, CH_3_), 1.03 (s, 3H, CH_3_); ^13^C NMR (100 MHz, CDCl_3_): *δ* = 190.9, 141.0, 134.8, 131.0, 129.5, 128.3, 115.3, 91.1, 47.1, 42.7, 31.6, 31.5, 29.1, 28.2, 27.8, 11.3; LC/MS (ESI): 463 [M]^+^; Anal. for C_23_H_30_ClN_3_O_5_; calcd: C, 59.54; H, 6.52; Cl, 7.64; N, 9.06; Found: C, 59.57; H, 6.51; Cl, 7.60; N, 9.02.

#### 5-((4-Bromophenyl)(2-hydroxy-4,4-dimethyl-6-oxocyclohex-1-en-1-yl)methyl)-2,6-dioxo-1,2,3,6-tetrahydropyrimidin-4-olate (4o)

**4n** was prepared from barbituric acid **1a**, dimedone **2** and *p*-bromobenzaldehyde **3** according to the general procedure (**GP1**) yielding a white solid material (678 mg, 1.33 mmol, 89%). m.p: 208°C; IR (KBr, *cm*^*–1*^): 3093, 2939, 2885, 2829, 2551, 1746, 1686, 1576, 1506, 1466, 1416, 1268, 1241; ^1^H NMR (400 MHz, CDCl_3_): *δ* 13.31 (s, 1H, OH), 8.67 (brs, 2H, NH), 7.05 (m, 4H, Ph), 5.79 (s, 1H, benzyl-H), 2.79(q, 4H, *J* = 7.3 Hz, C*H*_2_CH_3_), 2.35 (d, 4H, *J* = 5.1 Hz, CH_2_), 1.21(t, 6H, *J* = 7.3 Hz, CH_2_C*H*_3_), 1.11 (s, 3H, CH_3_), 1.03(s, 3H, CH_3_); ^13^C NMR (100 MHz, CDCl_3_): *δ* = 198.5, 180.1, 152.8, 140.5, 131.4, 130.7, 128.7, 128.6, 118.5, 115.6, 91.0, 50.9, 42.8, 31.6, 31.5, 29.2, 28.3, 27.8, 11.3; LC/MS (ESI): 508 [M]^+^; Anal. for C_23_H_30_BrN_3_O_5_; calcd: C, 54.34; H, 5.95; Br, 15.72; N, 8.27; Found: C, 54.35; H, 5.96; Br, 15.69; N, 8.30.

#### 5-((2-Hydroxy-4,4-dimethyl-6-oxocyclohex-1-en-1-yl)(naphthalen-2-yl)methyl)-2,6-dioxo-1,2,3,6-tetrahydropyrimidin-4-olate (4p)

**4q** was prepared from barbituric acid **1a**, dimedone **2** and 2-naphthaldehyde **3** according to the general procedure (**GP1**) yielding an oily product (646 mg, 1.35 mmol, 90%). IR (KBr, *cm*^*–1*^): 3049, 2948, 2863, 2725, 1685, 1594, 1508, 1371, 1252, 1216; ^1^H NMR (400 MHz, CDCl_3_): *δ* 14.25 (s, 1H, OH), 7.46-7.22 (m, 7H, naphthyl), 6.21 (s, 1H, benzyl-H), 3.27 (s, 6H, 2CH_3_), 3.25 (s, 6H, 2CH_3_), 3.14(q, 4H, *J* = 7.3 Hz, C*H*_2_CH_3_), 2.41 (q, 4H, *J* = 5.1 Hz, CH_2_), 2.23 (s, 2H, CH_2_), 1.37 (t, 6H, *J* = 7.3 Hz, CH_2_C*H*_3_), 1.07(s, 3H, CH_3_), 1.01 (s, 3H, CH_3_); ^13^C NMR (100 MHz, CDCl_3_): *δ* = 199.1, 180.5, 165.5, 164.2, 152.5, 149.7, 136.8, 131.5, 129.9, 126.5, 124.2, 115.5, 114.7, 89.9, 50.9, 45.5, 41.7, 31.3, 30.7, 28.2, 11.3; LC/MS (ESI): 479 [M]^+^; Anal. for C_27_H_33_N_3_O_5_; calcd: C, 67.62; H, 6.94; N, 8.76; Found: C, 67.65; H, 6.96; N, 8.80.

## Competing interests

The authors declare that they have no competing interests.

## Authors’ contributions

AB proposed the subject, designed the study. AMA carried out the synthesis of all the products. YNM and AMA helped in the results and discussion. MRHS carried out NMR spectroscopy and elemental analysis. HG and HKF carried out the X-ray crystallography part. AB prepared draft the manuscript. All the authors read and approved the final manuscript.
